# Upregulation of oncogenic MUC1 by the EMT transcription factor brachyury mediates immunotherapy-resistance in tumor cells

**DOI:** 10.1186/2051-1426-3-S2-P294

**Published:** 2015-11-04

**Authors:** Justin M David, Duane H Hamilton, Charli L Dominguez, Jeffrey Schlom, Claudia Palena

**Affiliations:** 1 Laboratory of Tumor Immunology and Biology, Center for Cancer Research, National Cancer Institute, National Institutes of Health, Bethesda, MD, USA

## 

Epithelial-mesenchymal transition (EMT) is a molecular and cellular program in which epithelial cells lose their well-differentiated phenotype and adopt mesenchymal characteristics. This process occurs during the progression of cancer to metastatic disease, and is also associated with recurrence as conventional therapies fail to eliminate the minority of resistant tumor cells that have undergone EMT. Brachyury is a transcription factor that controls EMT during development, and we have shown that brachyury is aberrantly overexpressed in various human cancers where it promotes tumor cell EMT, metastatic dissemination, and resistance to conventional therapies. Further, we have recently reported that very high expression of brachyury can protect against caspase-dependent immune cell-mediated cytotoxicity.

In seeking to elucidate mechanisms of immunotherapy resistance, we have discovered a novel association between brachyury and mucin-1 (MUC1). MUC1 is overexpressed in the majority of carcinomas, and it has been shown to mediate oncogenic signaling, induce EMT, increase stem cell characteristics, and confer resistance to genotoxic agents. Here, we report a novel positive association between brachyury and MUC1 in both cancer cell lines and patient tumor tissues, in which brachyury was found to upregulate MUC1 expression at the level of transcript stability. Targeting MUC1 by siRNA-based gene silencing was found to abrogate the resistance of brachyury-expressing tumor cells to killing by recombinant tumor necrosis-related apoptosis-inducing ligand (TRAIL) and to lysis by natural killer (NK) cells. These studies confirm a protective role for MUC1 as a mediator of immunotherapy-resistance in brachyury-expressing tumor cells, and demonstrate that targeting MUC1 restores the susceptibility of mesenchymal tumor cells to immune attack.

